# Lesion-conditioning of synthetic MRI-derived subtraction-MIPs of the breast using a latent diffusion model

**DOI:** 10.1038/s41598-024-56853-1

**Published:** 2024-03-16

**Authors:** Lorenz A. Kapsner, Lukas Folle, Dominique Hadler, Jessica Eberle, Eva L. Balbach, Andrzej Liebert, Thomas Ganslandt, Evelyn Wenkel, Sabine Ohlmeyer, Michael Uder, Sebastian Bickelhaupt

**Affiliations:** 1 Institute of Radiology, Uniklinikum Erlangen, Friedrich-Alexander-Universität Erlangen-Nürnberg (FAU), Maximiliansplatz 3, 91054 Erlangen, Germany; 2 Chair of Medical Informatics, Friedrich-Alexander-Universität Erlangen-Nürnberg (FAU), Wetterkreuz 15, 91058 Erlangen-Tennenlohe, Germany; 3 Pattern Recognition Lab, Friedrich-Alexander-Universität Erlangen-Nürnberg (FAU), Martensstraße 3, 91058 Erlangen, Germany; 4 Radiologie München, Burgstraße 7, 80331 Munich, Germany

**Keywords:** Translational research, Magnetic resonance imaging, Breast cancer, Cancer imaging, Image processing, Machine learning

## Abstract

The purpose of this feasibility study is to investigate if latent diffusion models (LDMs) are capable to generate contrast enhanced (CE) MRI-derived subtraction maximum intensity projections (MIPs) of the breast, which are conditioned by lesions. We trained an LDM with n = 2832 CE-MIPs of breast MRI examinations of n = 1966 patients (median age: 50 years) acquired between the years 2015 and 2020. The LDM was subsequently conditioned with n = 756 segmented lesions from n = 407 examinations, indicating their location and BI-RADS scores. By applying the LDM, synthetic images were generated from the segmentations of an independent validation dataset. Lesions, anatomical correctness, and realistic impression of synthetic and real MIP images were further assessed in a multi-rater study with five independent raters, each evaluating n = 204 MIPs (50% real/50% synthetic images). The detection of synthetic MIPs by the raters was akin to random guessing with an AUC of 0.58. Interrater reliability of the lesion assessment was high both for real (Kendall’s W = 0.77) and synthetic images (W = 0.85). A higher AUC was observed for the detection of suspicious lesions (BI-RADS $\ge$ 4) in synthetic MIPs (0.88 vs. 0.77; p = 0.051). Our results show that LDMs can generate lesion-conditioned MRI-derived CE subtraction MIPs of the breast, however, they also indicate that the LDM tended to generate rather typical or ‘textbook representations’ of lesions.

## Introduction

Imaging plays a pivotal role in the initial diagnosis, treatment, and follow-up of breast cancer. Among available methods, magnetic resonance imaging (MRI) has been described as the most sensitive modality for detecting suspicious abnormalities within the breast tissue^1–4^. Breast MRI commonly includes a multiparametric set of sequentially acquired image contrasts. Contrast enhanced (CE) series include T1-weighted sequences that are acquired before and after the administration of contrast agents. These can be used to derive subtraction series to visualize the mere perfusion component of the tissue, which is commonly altered in suspicious areas or focal lesions. The volumetric information about CE areas of the subtraction series can be condensed into a 2-dimensional (2D) image by computing maximum intensity projections (MIP). Those 2D MIPs have been increasingly investigated in abbreviated breast MRI reading protocols as a potential primary assessment, e.g. to be used in high-throughput settings of breast MRI such as screening of women with dense breast tissue and/or high-risk patients^5–7^.

Deep Learning (DL) is becoming increasingly important for the automated processing of medical images. Recently, DL has also been applied to characterize lesions in breast MRI^8,9^. However, there still remains the issue of the limited size of well-annotated datasets^10^. The relative low number of cases compared to other areas such as chest X-ray classification limits the performance of neural networks (NN). Generative NNs based on the diffusion process recently received great attention for the generation of natural appearing images using only text inputs^11,12^. Their goal is to generate new images that can either be unconditional or conditioned on, e.g., text, classes, or segmentation masks^11^. *Latent diffusion models* (LDM) can be trained efficiently in a lower dimensional “latent space” instead of the high-dimensional pixel-space^11^. Recently, they have also been applied to medical datasets, such as chest X-rays and brain MRI^13–16^, mostly with the goal to augment datasets for the training of NNs.

In the context of breast MRI, LDMs have been applied, for example, by Khader et al., to pre-train a NN with synthetic data to improve segmentation performance^15^. The group of Graham et al. developed Diffusion denoising probabilistic models (DDPM) for out-of-distribution detection and evaluated them on a medical dataset that included also breast MRI data^17^. Our feasibility study aims at investigating the capability of LDMs to generate synthetic MRI-derived CE-MIPs of the breast that are conditioned by lesions.

## Results

### Study Sample Characteristics

A total of n = 1966 patients (median age at first examination [IQR]: 50 [IQR: 42 to 59] years) with a total of n = 2832 breast MRI examinations were included in this analysis. Multiple examinations were performed in n = 495 patients (Table 1). The autoencoder NN was trained with all available examination MIPs whereas the LDM was conditioned on a subset thereof for which segmentations were available. The training dataset for conditioning the LDM with the segmented lesions contained n = 407 examination MIPs of n = 338 patients (median age [IQR]: 50 [IQR: 41.50 to 59] years) with a total of n = 756 lesions. The validation dataset consisted of n = 102 examination MIPs of n = 84 patients (n = 193 lesions). According to the histopathology, 160 out of 407 MIPs (39%)in the training dataset contained malignant lesions, whereas in the validation dataset, 37 out of 102 MIPs (36%) contained malignant lesions. Details on the conditioning subset are given in Table 2.

**Table 1:** Cohort used for training the auto-encoder.

Variable	Cohort
N examinations	2832
N patients	1966
Median age (IQR), first examination [years]	50 (42–59)
N repeated examinations per patient	
One examination	1471
Two examinations	281
Three examinations	108
Four examinations	61
Five examinations	41
Six examinations	3
Eight examinations	1

**Table 2:** Cohort used for training and validating the latent diffusion model. Percentages of the histopathology results are given in relation to all available cases that were classified with the respective BI-RADS score, including those for which the pathology reports were not available. *No.* number of, *N/A* not available histopathology results.

Variable	Diffusion subset training	Diffusion subset validation
N examinations	407	102
N patients	338	84
Median age (IQR) [years]	50 (41.50–59)	49 (42–58.75)
N repeated examinations per patient		
One examination	291	72
Two examinations	33	8
Three examinations	7	3
Four examinations	6	0
Five examinations	1	1
Maximum BI-RADS scores (%)		
BI-RADS 1	117/407 (28.75)	31/102 (30.39)
BI-RADS 2	73/407 (17.94)	17/102 (16.67)
BI-RADS 3	11/407 (2.70)	3/102 (2.94)
BI-RADS 4	71/407 (17.44)	18/102 (17.65)
BI-RADS 5	34/407 (8.35)	12/102 (11.76)
BI-RADS 6	101/407 (24.82)	21/102 (20.59)
No. of cases with histopathology-proven malignance per MIP (%)		
BI-RADS 3	0/11 (0%; N/A: n = 0)	1/3 (33.3%; N/A: n = 0)
BI-RADS 4	28/63 (39.4%; N/A: n = 8)	7/15 (38.9%; N/A: n = 3)
BI-RADS 5	31/33 (91.2%; N/A: n = 1)	8/10 (66.7%; N/A: n = 2)
BI-RADS 6	101/101 (100%; N/A: n = 0)	21/21 (100%; N/A: n = 0)
No. of lesion instances per MIP [min/mean (sd)/max]	1/2.61 (± 2.11)/11	1/2.72 (± 2.25)/10

### Diffusion model outputs

**Figure 1:**
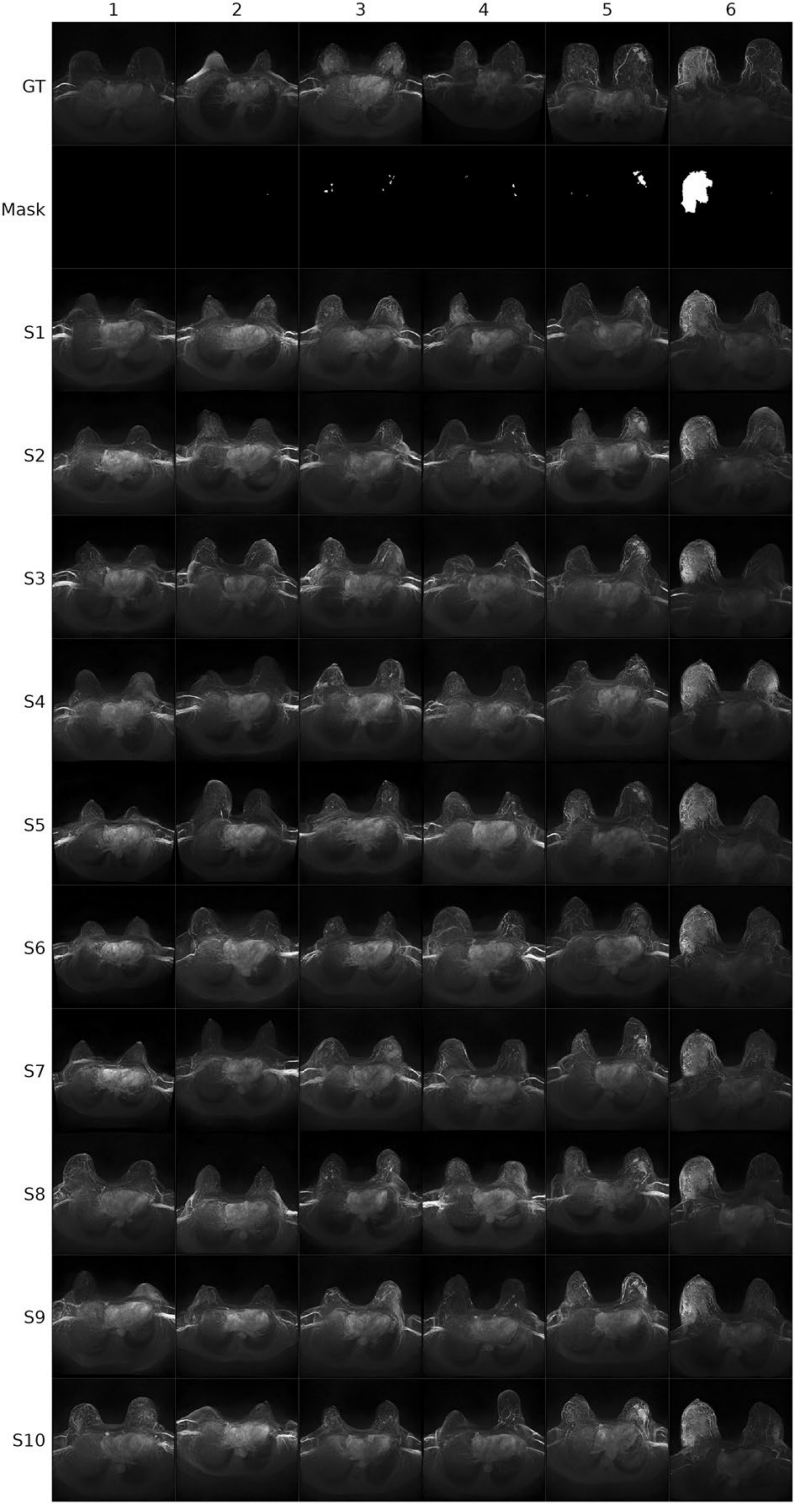
Example images (A) for six cases (BI-RADS 1–6). Row 1 (*GT* ground truth) shows the acquired breast MRI data with the contrast enhanced maximum intensity projection (MIP) depicted. Row 2 shows the segmentation mask of the lesion from the GT, which was used for conditioning the latent diffusion model. Rows 3–12 show generated synthetic example images (S1–S10). For each BI-RADS class one example image is given in the figure (columns). *GT* ground truth, *BI-RADS* breast imaging reporting and data system.

**Figure 2:**
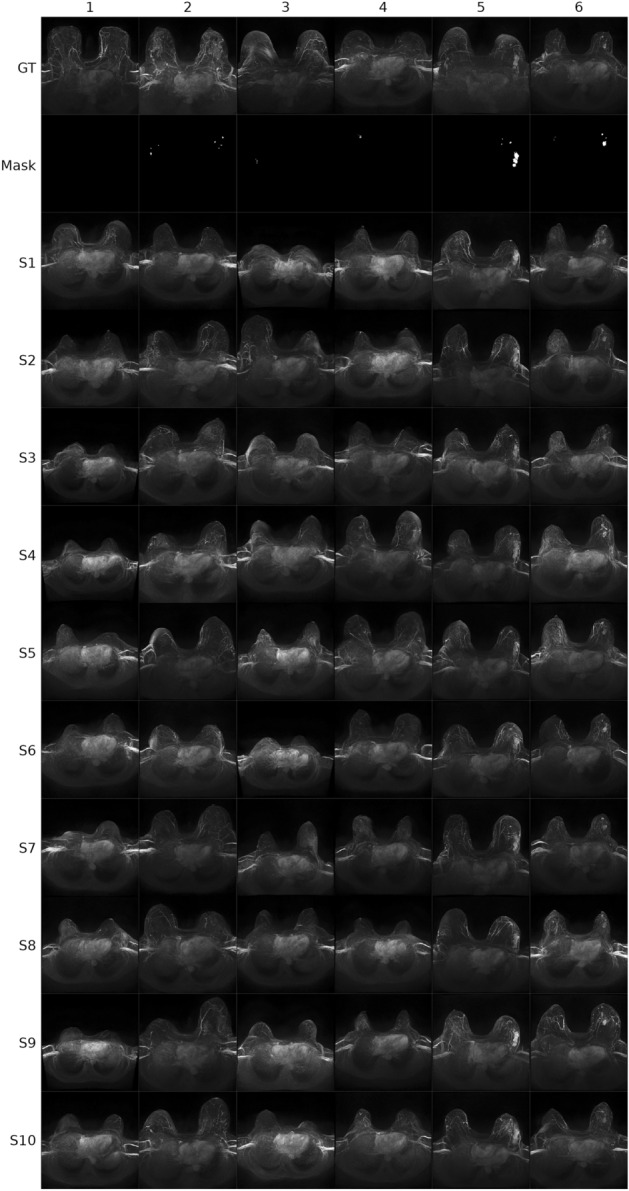
Example images (B) for six cases (BI-RADS 1–6). Row 1 (*GT* ground truth) shows the acquired breast MRI data with the contrast enhanced maximum intensity projection (MIP) depicted. Row 2 shows the segmentation mask of the lesion from the GT, which was used for conditioning the latent diffusion model. Rows 3–12 show generated synthetic example images (S1–S10). For each BI-RADS class one example image is given in the figure (columns). *GT* ground truth, *BI-RADS* breast imaging reporting and data system.

The model weights from the epoch with the lowest validation loss (epoch 376) were used to generate the synthetic MRI-derived CE-MIPs of the breast. The training- and validation loss curves of the conditioned LDM are given in supplement S5. For demonstration purposes, n = 120 examples of the generated synthetic breast MRI MIPs as well as the segmentation masks used for conditioning the LDM and the corresponding acquired MRI data (GT) are given in Figs. 1 and 2. Regarding sampling diversity, the average Multi-scale structural similarity metric (MS-SSIM)^18^ between the n = 10 synthetic MIPs and corresponding real MIP per case in the validation dataset was 0.533 ($\pm \hspace{0.17em}$0.09) on average. FID was 0.215, computed with all n = 1020 synthetically generated images (10 per segmentation mask) and the corresponding n = 102 real MIP images from the validation dataset. In comparison, FID among real images was < 0.001.

### Conditioning evaluation

#### Reading task 1: lesion assessment

Kendall’s coefficient of concordance (W) as a measure of interrater reliability of the lesion assessment task was W = 0.77 (p = < 0.001) for real MIPs and W = 0.85 (p = < 0.001) for synthetic images (corresponding to ‘substantial’ and ‘almost perfect’ agreements). The Wilcoxon test showed no significant differences in the lesion assessment between real MIPs (median [IQR]: 2.67 [IQR: 2.00 to 3.92]) and synthetic MIPs (median [IQR]: 2.67 [IQR: 2.00 to 4.25]) (p = 0.650).

**Figure 3:**
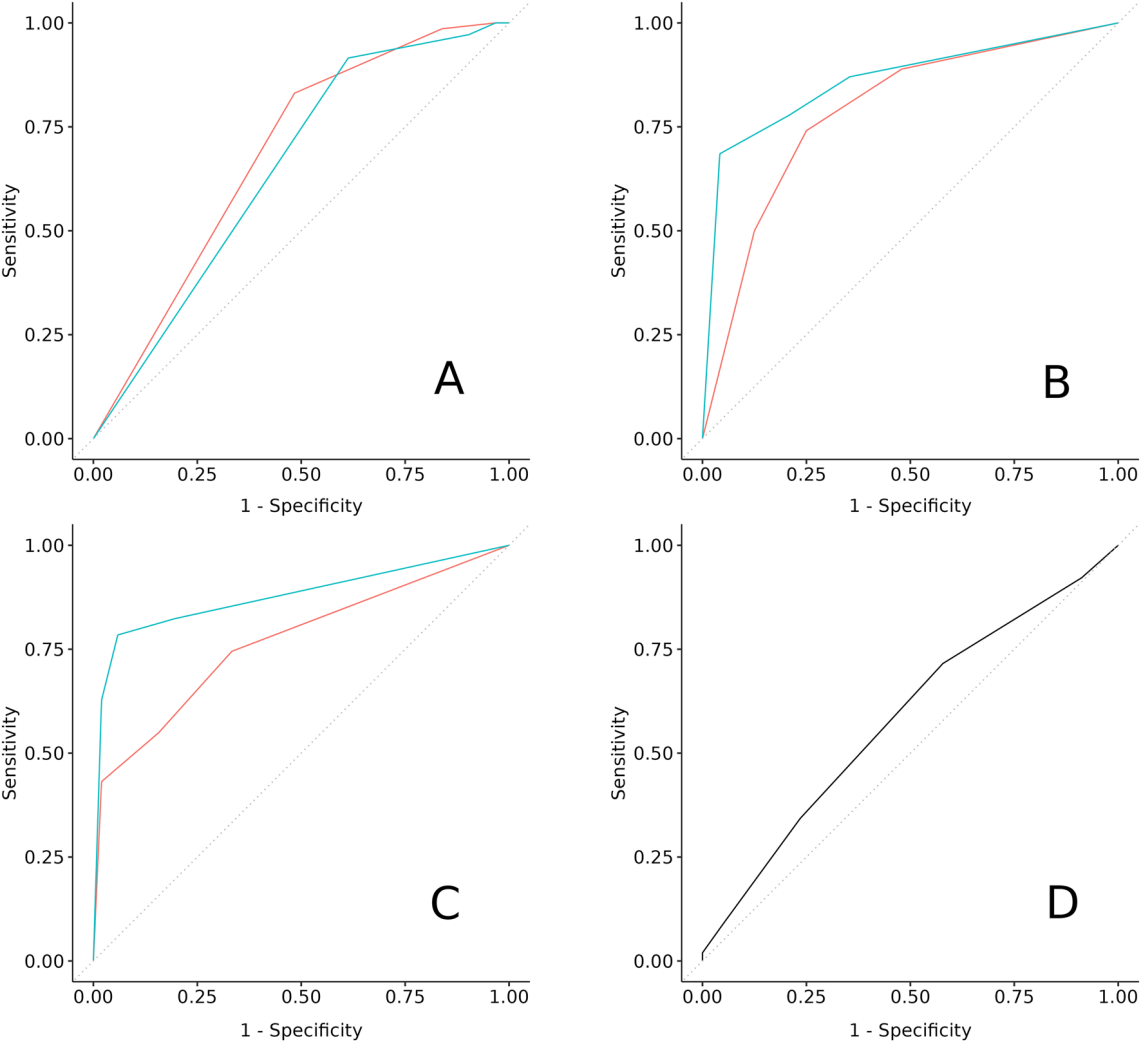
Receiver operating characteristic (ROC) curves of reading tasks 1 and 2. (**A**) Detection of any lesions (BI-RADS > = 2). (**B**) Detection of potentially significant lesions (BI-RADS > = 3). (**C**) Detection of suspicious lesions (BI-RADS > = 4). (**D**) Detection of synthetic MIPs. Red line: real images. Blue line: synthetic images. *BI-RADS* breast imaging reporting and data system, *MIP* maximum intensity projection.

Fleiss’ Kappa computed as a measure of interrater agreement for the derived binarized outcome *presence of any lesions* (BI-RADS $\ge$ 2) was Kappa = 0.13 (p = 0.024) for real MIPs and Kappa = 0.23 (p = < 0.001) for synthetic images (corresponding to ‘slight’ and ‘fair’ agreements). The area under the receiver operating characteristics (ROC) curve (AUC) for the detection of *any lesions* in real MIPs was 0.68 (for the results of the individual raters please refer to supplement S7), whereas in synthetic MIPs the AUC was 0.65 (Fig. 3A) (for the results of the individual raters please refer to supplement S7). DeLong’s test showed no significant differences regarding the detection of *any lesions* in real and synthetic images (p = 0.635). Columns 2–4 of Table 3 show the corresponding contingency table.

**Table 3:** Confusion matrix of the combined interrater label vs. ground truth, stratified by real and synthetic images, for any lesions (BI-RADS >= 2, columns 2–4), potentially significant lesions (BI-RADS >= 3, columns 5–7), and suspicious lesions (BI-RADS >= 4, columns 8–10). *GT* ground truth (defined as described in the “Methods”), *BI-RADS* breast imaging reporting and data system, *MIP* maximum intensity projection.

	Reading	BI-RADS > = 2			BI-RADS > = 3			BI-RADS > = 4		
		GT 0	GT 1	Total	GT 0	GT 1	Total	GT 0	GT 1	Total
Real MIPs	0	5	1	6	36	14	50	43	23	66
	1	26	70	96	12	40	52	8	28	36
	Total	31	71	102	48	54	102	51	51	102
Synthetic MIPs	0	3	2	5	38	12	50	48	11	59
	1	28	69	97	10	42	52	3	40	43
	Total	31	71	102	48	54	102	51	51	102

For the *presence of potentially significant lesions* (BI-RADS $\ge$ 3), the interrater agreement was Kappa = 0.5 (p = < 0.001) for real MIPs and Kappa = 0.67 (p = < 0.001) for synthetic images (corresponding to ‘moderate’ and ‘substantial’ agreements). The AUC for the detection of *potentially significant lesions* in real MIPs was 0.79 (see supplement S7), whereas in synthetic MIPs the AUC was 0.86 (Fig. 3B) (for the results of the individual raters please refer to supplement S7). DeLong’s test showed no significant differences regarding the detection of *potentially significant lesions* in real and synthetic images (p = 0.205). Columns 5–7 of Table 3 show the corresponding contingency table.

With respect to the derived binarized outcome *suspicious lesions* (BI-RADS $\ge 4$), the interrater agreement was Kappa = 0.55 (p = < 0.001) for real MIPs and Kappa = 0.74 (p = < 0.001) for synthetic images (corresponding to ‘moderate’ and ‘substantial’ agreements). The AUC for detecting *suspicious lesions* in real MIPs was 0.77 (for the results of the individual raters please refer to supplement S7), whereas in synthetic MIPs the AUC was 0.88 (Fig. 3C) (for the results of the individual raters please refer to supplement S7), corresponding to a not significant difference according to DeLong’s test (p = 0.051). Columns 8–10 of Table 3 show the corresponding contingency table. More information on the interrater agreements between individual raters as well as the conditioning evaluation on a per rater level are given in supplements S6 and S7.

#### Reading task 2: detection of synthetic MIPs

The interrater agreement in the detection of synthetic MIPs was Kappa = − 0.009 (p = 0.682). The contingency table of the combined interrater label and the ground truth (GT) is shown in Table 4. The false negative rate in detecting synthetic MIPs was 66% (67/102) with a specificity of 76% (78/102). The AUC for the detection of synthetic MIPs was 0.58 (Fig. 3D) (for the results of the individual raters please refer to supplement S8). Both, the low interrater agreement (‘poor’) and the ROC curve indicate that the detection of synthetic MIPs is akin to random guessing. More details regarding the interrater agreements in between individual raters are given in supplement S6.

**Table 4:** Confusion matrix of the reading vs. ground truth of the task 2 to decide for each maximum intensity projection (MIP) if it is a real MIP (‘0’) or a synthetic MIP (‘1’). GT: ground truth, i.e. if the image was a real MIP or synthetically generated by the latent diffusion model.

Reading	GT 0	GT 1	Total
0	78	67	145
1	24	35	59
Total	102	102	204

#### Reading tasks 3: anatomical correctness

**Figure 4:**
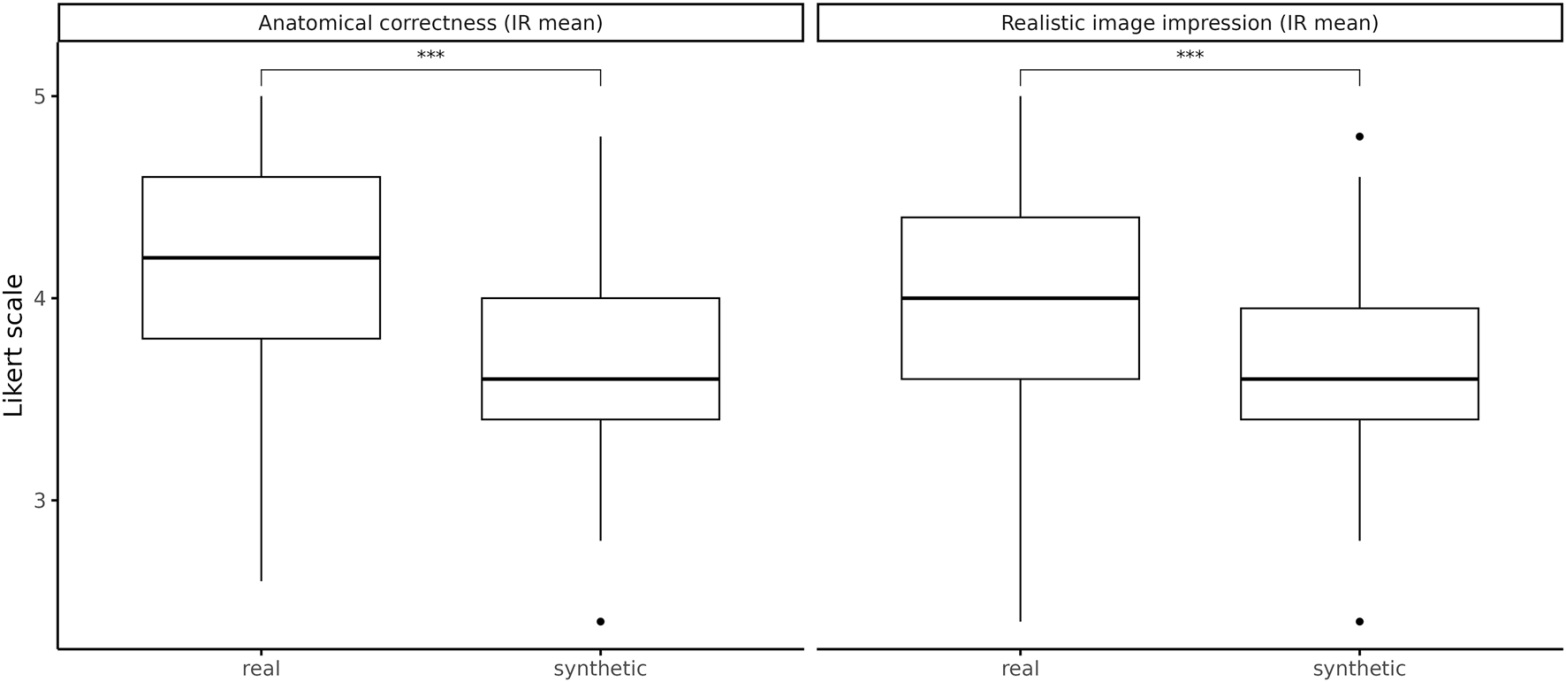
Boxplots of the interrater (IR) label to describe the arithmetic mean between the raters of the Likert-scaled readings ‘anatomical correctness’ (task 3) and ‘realistic image impression’ (task 4). Likert-scale: 1—strongly disagree; 2—disagree; 3—neither agree nor disagree; 4—agree; 5—strongly agree. *ns* not significant. ***p-value of the Wilcoxon Rank Sum Test < 0.001.

The interrater reliability in the scoring of anatomical correctness was W = 0.33 (p = < 0.001) for real MIPs and W = 0.24 (p = 0.084) for synthetic images (both corresponding to ‘fair’ agreements). Figure 4 (left) shows a significantly lower anatomical correctness of synthetic MIPs in the Wilcoxon test (median [IQR]: 3.60 [IQR: 3.40 to 4.00]) as compared to real MIPs (median [IQR]: 4.20 [IQR: 3.80 to 4.60]) (p = < 0.001).

#### Reading tasks 4: realistic image impression

The interrater reliability when scoring realistic image impression was W = 0.3 (p = < 0.001) for real MIPs and W = 0.21 (p = 0.291) for synthetic images (both corresponding to ‘fair’ agreements). Figure 4 (right) shows a significantly lower realistic image impression of synthetic MIPs in the Wilcoxon test (median [IQR]: 3.60 [IQR: 3.40 to 3.95]) as compared to real MIPs (median [IQR]: 4 [IQR: 3.60 to 4.40]) (p = < 0.001). More details regarding the individual raters’ results for reading tasks 3 and 4 are given in supplements S6 and S9.

## Discussion

This study demonstrates an LDM that generates synthetic CE subtraction MIPs. The LDM was trained with n = 2832 CE-MIPs of the breast of n = 1966 patients. The conditioning process of the LDM was performed with n = 756 segmented lesions that indicated the underlying BI-RADS class and location, thus implicitly providing information on morphometric characteristics of these lesions. With an AUC of 0.58, the images generated by the LDM were not distinguishable from actual MRI-acquired data by five independent raters. The low MS-SSIM value found in our evaluation might be an indicator supporting this assessment, suggesting that the LDM generates synthetic images with a high diversity when sampling multiple images from the same segmentation mask being used for the conditioning.

According to our multi-rater study, the detection of synthetic MIPs was akin to random guessing. Nevertheless, we found that there may also be a certain training effect in recognizing synthetically generated medical images: R1, R2, R3 and R5 had, according to their own statements, no previous experience with synthetically generated subtraction MIPs of the breast, whereas R4 as the medical supervisor of the experiments for this study has evaluated and reviewed LDM-generated MIPs of the breast already before. This previous experience is also evident in the AUC of 0.64 that was achieved by R4 in detecting synthetic MIPs (see supplement S8). Furthermore, no differences could be observed in the detection of *any lesions* (BI-RADS $\ge$ 2). These observations suggest that the amount of training data was sufficient to condition the LDM with lesions. The multi-rater study further showed that both the detection of *potentially significant lesions* and especially *suspicious lesions* tended to be better in the synthetic data, however, not reaching statistical significance. This holds true for both the combined interrater labels and on an individual rater level, indicating that the LDM may have learned rather typical or ‘textbook representations’ of (suspicious) lesions, whereas cases in the acquired MRI data apparently seemed not to be as consistently assignable to the underlying class in our multi-rater study.

We hypothesize that this finding may be related to the manner in which the LDM was conditioned with the segmentations and that larger amounts of training data probably might not remedy this. We assume that there is some heterogeneity in the visual appearance of lesions of a specific BI-RADS class on CE-MIPs with potential overlaps between lesions of different classes (see schema in supplement S10). However, the GT of the lesions depicted on the MIPs was established using the clinical reports, which were based on the full diagnostic multiparametric protocol that contained much more information than visible in the MIPs. As this additional information was naturally lacking during the LDM training, the NN might have inferred general patterns between heterogeneous appearing lesions of the same class. These patterns may be reflected insofar as the NN, when generating synthetic data, could tend to generate lesions that can be assigned more clearly to a particular class. Thus, the LDM might have learned to represent lesions in some ranges with a higher confidence, as reflected by the non-overlapping regions in the distribution curves of lesion appearance from the schema in supplement S10. This might explain the observed differences in the detection of (suspicious) lesions between synthetic and actual MRI-derived breast CE-MIPs.

We consider this finding relevant as it indicates the requirement for future research to, first, investigate if this potential limitation can be overcome by more sophisticated conditionings, and second, to further elucidate the effects when using synthetically generated ‘textbook-alike’ data in potential areas of application such as the augmentation of training data for medical imaging DL tasks.

This feasibility study has several limitations. First, although not statistically significant, according to the multi-rater study the detection of lesions tended to be better in the synthetic data, pointing towards a confined capability of the trained LDM to generate a dataset that mimics the properties of lesions contained in an actual clinical breast CE-MIP dataset. Future research is required to investigate how the training of LDMs could be improved to better reflect the full spectrum of real-world lesions emphasizing the necessity to represent the diversity of indiscriminate lesions and overcoming the limitation of benefiting from ‘textbook representations’ during the training process. For example, the conditioning of the lesions could be extended by confidence measures, e.g., reflecting the degree of agreement between multiple raters, or to divide the defined classes into finer segments and explicitly annotate edge cases as such. Furthermore, LDMs could be conditioned with more parameters, including, a greater variety of clinical findings and anatomical heterogeneity, different grades of image quality, breast density, background parenchymal enhancement, scanner related features and the full multiparametric spectrum of breast MRI sequences to enable a more detailed property adjustment when generating synthetic breast MRI datasets.

Second, the fact that our reading study was also performed by two inexperienced raters (R1 and R3) could be used as an argument to question the validity of the results of this study, especially with regard to reading task 1 to categorize breast lesions, which, was performed by a medical research assistant, a breast MRI-experienced resident, and additionally by one board certified radiologist (whereas reading tasks 2–4 were performed by three board certified radiologists alongside the two inexperienced raters). Especially regarding the categorization of breast lesions (reading task 1), we believe that the expressiveness of our results might even benefit from the reading by the more inexperienced raters. While inexperienced raters could certainly have difficulty distinguishing between edge cases, especially when reading MIPs as the only source of information, their performance could also be used as a proxy to make certain assumptions about the representation of findings in the images. So, if hypothetically an inexperienced rater would be able to distinguish better between different classes on a synthetically generated image than on a real image, this could suggest that the process to generate the synthetic images has some properties that results in a representation of classes that allows even the inexperienced rater to distinguish them. For the lesion categorization (reading task 1), the substantial and almost perfect individual interrater agreements between both of the inexperienced raters and the board certified radiologist (see supplement S6) allowed us to further assess the as such labeled images to get a deeper understanding of the LDM’s generative capabilities. With regard to the individual readings of the lesion categorization, no significant differences were observed in the detection of *any lesions* and *potentially significant lesions* between real and synthetic MIPs for all individual raters (see paragraphs 1–3 of supplement S7). Regarding the detection of *potentially significant lesions*, the ROC-curves of the individual raters shown in supplemental Figure Supp. 3 further indicate that, although not significantly different, regardless of the raters’ experience, the AUC was constantly higher for the detection in synthetic MIPs (blue curves) as compared to real MIPs (red curves). With respect to *suspicious lesions*, while no significant differences between real and synthetic images could be observed for R1 and R2, R3 was able to significantly better detect these lesions on the synthetic MIPs according to DeLong’s test for two ROC curves (AUC: 0.86 vs. 0.69, p = 0.001) (see paragraph 3 of supplement S7 and Figure Supp. 4). The trend of a better lesion characterization on synthetic MIPs, visible in the ROC-curves computed from the combined interrater labels (Fig. 3), is similar to the trend observed in the individual readings (supplemental figures Supp. 2, Supp. 3, and Supp. 4). Furthermore, the trend does not seem to depend on the raters’ experience, which further supports the validity of the methodological approach as well as the trustworthiness of these results. These results also show that when evaluating synthetic images, it seems to be important to review such images with regard to different aspects, i.e. by providing raters with tasks that focus on specific peculiarities of the images in order to be able to decipher their synthetic origin.

Third, the anatomical correctness and realistic image impression were scored significantly lower in the synthetic MIPs in our multi-rater study, indicating that the training of our autoencoder NN could have benefited from additional training data. However, our sample size is comparable to those reported, for example, by Kadher et al. (1250 knee MRI exams, 998 brain MRI exams, 1844 breast MRI exams, and 1010 lung CTs) using LDMs to generate volumetric medical datasets^15^. As a technical metric to describe how realistic the synthetic images are, we report the FID metric for our model on this dataset to serve as a potential benchmark for future developments. Future studies and evaluations could include as well extended direct comparisons of LDMs with other image generation techniques such as GAN-based approaches in the context of MRI-derived CE breast MIPs, which, however, was beyond the scope of this present study. Another limitation is that our experiments focused on 2D images with a lower resolution than being used in the clinical setting. To create even more realistic datasets, future works should train LDMs to generate high-resolution 3D volumes.

The application of LDMs for synthetic data generation in medical imaging is an emerging research area. For example, Pinaya et al. demonstrated the generation of brain MRI datasets conditioned by different anatomical parameters^13^. In another study, Khader et al. applied LDMs to generate computed tomography and MRI sequences of various anatomical regions^15^. The insufficient availability of annotated training data is often an important limitation to DL development in medical imaging^10^. As mentioned, for example, by^13^ and demonstrated by Khader et al.^15^, an obvious application of LDMs is the augmentation of medical imaging training datasets. For example, Khader et al. observed an improved segmentation performance when pre-training a NN with synthetic data that was generated by an LDM^15^. Herein, next to providing ad-hoc semantically enriched and large—in principle infinite—datasets, or the augmentation of certain rare cases to reduce bias in machine learning (ML), LDMs might enable as well to improve privacy-preserving approaches for ML algorithms. Thus, our results demonstrate the capability to create synthetic data fitted to a potential clinical high-throughput setting such as (supplemental) MRI in breast cancer screening, in which (a) ML might be of special relevance in the future and (b) large and representative datasets are important to reduce the potential bias of the algorithms. Nevertheless, as the synthetically generated datasets may contain restrictions that could potentially limit the generalizability of the therewith trained NNs, our results suggest that such an application of LDMs should currently be considered carefully, especially when being conditioned with only a limited set of parameters.

In conclusion, our study is among the first to demonstrate an LDM to generate synthetic MRI-derived CE-MIPs of the breast conditioned by lesions. Our multi-rater study further showed that the detection of (suspicious) lesions tended to be better in the synthetic data compared to actual MRI acquisitions, potentially indicating that the LDM might have generated ‘textbook representations’ of lesions in breast CE-MIPs. Further research is necessary to elucidate this finding and to investigate potential implications when using conditioned LDMs in medical imaging.

## Methods

### Study sample

This retrospective analysis was approved by the ethics committee of the Friedrich-Alexander-University (FAU) Erlangen-Nürnberg, which waived the need for written informed consent. The authors declare that this research was performed in compliance with the World Medical Association Declaration of Helsinki on Ethical Principles for Medical Research Involving Human Subjects. The study period was between October 2015 and June 2020. Within this period, female patients with a clinically indicated breast MRI performed with a full diagnostic protocol including CE sequences at the Institute of Radiology of the University Hospital Erlangen (UHE) were included in this study. The study sample is partially overlapping with previously reported cohorts in which (a) the automated detection of MRI artifacts on breast CE-MIPs by applying DL methods^19^ and (b) a DL-based image quality assessment in high b-value diffusion-weighted breast MRI^20^ were evaluated, as well as (c) an investigation of the prevalence of MRI-artifacts in breast CE-MIPs^21^. Details on the MRI protocols are given in supplement S1.

### Imaging data annotation

The examination MIPs used for training the LDM were annotated by a board certified radiologist. The MIPs were imported into the Slicer-3D Software^22^ and the presence of lesions was determined visually and by using the information available from the clinical reports. If no lesion was visible on the MIP it was classified with a BI-RADS score of “1”. Otherwise, all visible lesions were segmented accordingly (including the possibility that several lesions with different BI-RADS scores could be visible in one single MIP image). During the annotation, the radiologist had access to all clinical information including all sequences of the corresponding MRI study as well as the radiology and pathology reports.

### Deep learning

The LDM of this work builds upon the works of Rombach et al.^11^ and Esser et al.^23^. LDMs are a subgroup of generative models that are based on DDPMs, which are probabilistic NNs that aim at learning a data distribution by a successive perturbation of a normally distributed variable^24,25^. These models can be applied to generate new images that can either be unconditional or conditioned on, e.g., text, classes, or segmentation masks^11^. Regarding conditioned LDMs, to generate a new sample, first a random vector is created with size equal to the requested image. Then the NN gradually denoises this random vector with additional guidance of the conditioning. After a finite amount of steps, the final image is generated^11^. To reduce the computational effort, the above mentioned process is performed in the lower dimensional “latent space” instead of the high-dimensional pixel-space^11^. The representation of this latent space is achieved by an autoencoder NN, which has the purpose to reduce the dimensions of the input by compressing the information contained in the images. The autoencoder is trained to perform an identity mapping on the images, but with a bottleneck at its center to achieve the compression of the information^11^. The NN at the heart of the LDM is a denoising UNet^26^ that utilizes the cross-attention mechanism^27^ to efficiently learn the conditioning from various input modalities^11^.

Details on the imaging data preprocessing for training the NNs are given in supplement S2. Details on our LDM trainings are given in supplement S3. First, we trained a vector quantization variational autoencoder model (using the default parameters as described in^11^) to learn the representation of breast CE-MIPs in the *latent space*. For the conditioning, the data for which segmentations were available were randomly split by 80% to 20% into a training dataset and an independent validation dataset. The learning rate ($\eta =1.2\times {10}^{-5}$) and batch size were optimized with a grid search. For all other parameters, the default settings^11^ were used. Model convergence was evaluated visually using the training and validation loss curves and the training was stopped once no improvement was noticed on the validation set (see supplement S5). The trained LDM was used to sample 10 synthetic images of a resolution of $256\times 256$ pixels for every segmentation mask from the validation dataset, using 200 Denoising Diffusion Implicit Models steps.

### Diffusion model evaluation study

To assess the conditioning capabilities of the LDM, a multi-rater study was performed with five independent raters (R1: D.H., board certified radiologist with > 10 years of experience; R2: J.E., medical research assistant with > 2 years of experience; R3: E.L.B., radiologist resident with > 5 years of experience; R4: S.B., board certified radiologist with > 10 years of experience; R5: S.O., board certified radiologist with > 10 years of experience). This study consisted of four reading tasks in which the raters were asked to evaluate all n = 204 MIP images of the reading study dataset, consisting of the n = 102 MRI-derived breast CE-MIP images from the validation dataset as well as a set of n = 102 synthetic MIPs (randomly choosing one out of the n = 10 CE breast MRI MIPs that were generated by the LDM per case from the validation dataset). The raters were blinded to the proportion of real and synthetic MIPs in the reading study dataset. Using the segmentations from the validation dataset as inputs to the LDM for conditioning the synthetic images was intended to ensure that the synthetic dataset followed a similar data distribution in terms of lesion type and lesion size as the real MIPs in the validation dataset. The reading tasks were as follows:

1. Assess the presence and characteristics of lesions on the subtraction MIPs using BI-RADS analogue classifications (classes: 1—negative, 2—benign, 3—probably benign, 4—suspicious for malignancy, 5—highly suggestive of malignancy).
2. Classify each MIP into one of the two classes “real image” and “synthetic image”.
3. Assess on a 5-point Likert-scale for each MIP the question “How strongly do you agree with the following statement: ‘Anatomical structures are correctly represented on the MIP’”.
4. Assess on a 5-point Likert-scale for each MIP the question “How strongly do you agree with the following statement: ‘The image impression (texture, resolution, contrast) is realistic’”.

The raters were blinded to each other’s results. Initially, only the first reading task was communicated and for this task, the raters were additionally blinded to the fact that the dataset contained mixed real and synthetic MIPs. After completion of reading task 1, the raters were asked to complete reading tasks 2–4. Reading task 1 was performed by raters R1, R2, and R3, while reading tasks 2–4 were performed by all raters. The Likert-scale scoring was as follows: 1—strongly disagree; 2—disagree; 3—neither agree nor disagree; 4—agree; 5—strongly agree.

### Statistical analysis

Statistical measures were computed in the R programming language, version 4.2.2^28^. Interrater agreements were computed for the different outcomes of the reading tasks with the irr R package, version 0.84.1^29^, using Kendall’s coefficient of concordance^30^ to measure the interrater reliability of the ordinal scaled variables (BI-RADS analogue classifications, Likert-scales), and Fleiss’ Kappa^31,32^ to assess the interrater agreement of the binary outcomes. Interrater agreements are interpreted according to Landis and Koch^33^. To investigate the LDM’s generation capabilities regarding different aspects of the learned conditioning, binary labels were computed from each rater’s lesion assessment (reading task 1, lesion assessment according to the BI-RADS classification) in order to label the *presence of any lesions* (BI-RADS $\ge$ 2), the *presence of potentially significant lesions* (BI-RADS $\ge$ 3), and the *presence of suspicious lesions* (BI-RADS $\ge$ 4). For each image, those computed binary labels as well as the binary label from reading task 2 (detection of synthetic MIPs) were aggregated into final interrater labels each by calculating the arithmetic mean between the five raters in order to reflect the degree of agreement or confidence between the raters. Likewise, the Likert-scale based labels from reading tasks 3 and 4 (anatomical correctness and realistic image impression) were also aggregated into final combined labels by calculating the arithmetic mean between the raters for each image. To be able to analyze the binary interrater labels with contingency tables, images with an average interrater score $>0.5$ were considered to belong to the positive class (which corresponds to an aggregation according to the best-of-n method in the case of an uneven number of raters). Differences in the Likert-scaled ratings between real and synthetic MIPs were assessed with Wilcoxon’s rank sum test^34,35^. The lesion conditioning capabilities of the LDM were assessed with ROC curves, computed with the derived binary interrater labels and the corresponding ground truth (GT). Differences in the AUC between real and synthetic MIPs were assessed with DeLong’s test^36^. Multi-scale structural similarity metric (MS-SSIM)^18^ was computed using the implementation from the torchmetrics Python package, version 0.11.1^37^. MS-SSIM, with possible values between 0 and 1, was used in our study to evaluate the generation diversity with lower MS-SSIM values indicating a higher diversity (suggestive of “inventing” new images by the LDM) and higher values indicating the generation of more similar synthetic images. The MS-SSIM values were computed for each case in the validation dataset by generating 10 pairs each with the real MIPs and the 10 sampled synthetic images in order to assess the intra-case generation diversity. The reported value is the arithmetic mean across all accordingly computed MS-SSIM values from the cases of the validation dataset. Additionally, Fréchet Inception Distance (FID)^38^, using the implementation from the torchmetrics Python package, version 0.11.1^37^, was employed to assess if the generated synthetic images stem from a similar distribution as the original images. FID was computed using Inception v3 feature layer 64 and the original weights from^38^. The significance level was set to $\alpha$=0.05 for all statistical tests. No correction for multiplicity was performed. More details on the statistical analysis are given in the supplement S4.

### Supplementary Information

Supplementary Information.

## Data Availability

The datasets generated and/or analyzed during the current study are not publicly available due to internal data transfer policies but are available from the corresponding author on reasonable request.

## Code availability

The code used to perform the experiments is available at GitHub: https://github.com/kapsner/ldm-ce-mip-lesions.
