# Supplementary material for: Lesion-conditioning of synthetic MRI-derived subtraction-MIPs of the breast using a latent diffusion model
Source: Sci Rep. 2024 Mar 16;14:6391. doi: 10.1038/s41598-024-56853-1 (PMC10944528; doi:10.1038/s41598-024-56853-1)
Supplement: Supplementary file 1 — Supplementary Information. [file 41598_2024_56853_MOESM1_ESM.pdf]

Lesion-Conditioning of Synthetic MRI-Derived Subtraction-MIPs of the Breast Using a Latent Diffusion Model

Lorenz A. Kapsner[*,1,2,✉], Lukas Folle[*,3], Dominique Hadler[1], Jessica Eberle[1], Eva L. Balbach[1], Andrzej Liebert[1], Thomas Ganslandt[2], Evelyn Wenkel[4], Sabine Ohlmeyer[1], Michael Uder[1], and Sebastian Bickelhaupt[1,5]

[*] These authors contributed equally to this work.

[1] Institute of Radiology, Uniklinikum Erlangen, Friedrich-Alexander-Universität Erlangen-Nürnberg (FAU), Maximiliansplatz 3, 91054 Erlangen, Germany.

[2] Chair of Medical Informatics, Friedrich-Alexander-Universität Erlangen-Nürnberg (FAU), Wetterkreuz 15, 91058 Erlangen-Tennenlohe, Germany.

[3] Pattern Recognition Lab, Friedrich-Alexander-Universität Erlangen-Nürnberg (FAU), Martensstraße 3, 91058 Erlangen, Germany.

[4] Radiologie München, Burgstraße 7, 80331 München, Germany.

[5] German Cancer Research Center (DKFZ), Im Neuenheimer Feld 280, 69120 Heidelberg, Germany.

[✉] Correspondence: Lorenz A. Kapsner (M.D.) <lorenz.kapsner@uk-erlangen.de>

S1: MRI Protocols

All examinations were performed with the clinical routine MRI scanners (1.5 and 3.0 T MRI; Model names: Aera, Avanto, Skyra, Sola, and Vida; Siemens Healthineers, Erlangen, Germany). The clinical routine protocol includes an unenhanced T1-, T2-weighted, and diffusion-weighted imaging (DWI) sequences. Furthermore, a dynamic contrast-enhanced sequence is acquired after the intravenous administration of contrast agent. Similar to[1], this analysis was also performed with maximum intensity projections (MIPs) that were computed from the second postcontrast phase subtraction series (about 120 sec. after injection of contrast agent), which were created by the scanner systems. In case of a DIXON sequence, the in-phase images were used to compute the MIPs.

Table Supp. 1: The table contains the MRI parameter settings of the T1-weighted sequences that were related to the subtractions sequences used for creating the maximum intensity projections. *B_0 : magnetic field strength. T: Tesla. TE: echo time. TR: time to repetition. ms: millisecond. mm: millimeter. [1]: TE, TR, slice thickness not retrievable with DICOM tags '0018,0081', '0018,0080' and '0018,0050' for n=6 examinations. [2]: Acquisition Matrix not retrievable with DICOM tag '0018,1310' for n=26 examinations. [3], [4]: FoV not retrievable with DICOM tag '0051,100c' for n=581 examinations and n=1 examinations, respectively. N/A: not available. w/o: without. cond.: conditioning. tr.: training dataset. va.: validation dataset.*

**Model name**	**B_0 [T]**	**Sequence**	**Matrix**	**FoV [mm]**	**TE [ms]**	**TR [ms]**	**Slice thickness [mm]**	**N**	**N cond. tr.**	**N cond. va.**
Aera	1.5	FLASH (with DIXON)	384 × 323 to 448 × 376	379 - 420	4.77	6.49	1.5 - 1.6	324	30	5
		FLASH (w/o fat saturation)	384 × 336 to 448 × 394	340 - 429	4.77	7.7 - 7.72	1.7 - 2.1	63	3	5
Avanto	1.5	FLASH (w/o fat saturation)	448 × 331	340 - 399	4.78	7.58 - 8.32	1.5 - 1.8	631	164	40
Sola	1.5	FLASH (with DIXON)	448 × 358	379	4.77	6.5	1.6 - 1.8	2	0	0
Skyra fit	3.0	FLASH (with DIXON)	448 × 358 to 448 × 385	358 - 429	2.46	5.51 - 5.97	1.5 - 1.9	735	82	24

		VIBE (with DIXON)	448 × 385	359 - 399	2.46	5.97	1.5 - 1.7	74	30	5
		FLASH (w/o fat saturation)	448 × 385	359 - 399	2.46	6.04	1.5 - 1.7	55	16	8
Vida	3.0	FLASH (with fat saturation)	448 × 470	N/A	1.73	4.13	1.5	1[4]	0	0
		FLASH (with DIXON)	448 × 358 to 448 × 385	379	2.46	5.41	1.5 - 1.7	947[123]	82	15

S2: Imaging Data Preprocessing

All preprocessing steps were performed in Python (version 3.9.0) using the `SimpleITK` library, version 2.2.0[2,3]. The subtraction DICOM series were downloaded from our research XNAT[4] to the analytic workstation. MIPs were computed from the subtraction volume in direction of the z-axis. Resulting images were further resized to 256×256 pixels, rescaled to intensities between -1 and 1, and saved as `NumPy` arrays[5] that served as input for the neural networks (NNs). For the imaging data annotation, the computed MIPs were saved in the NIfTi file format.

S3: Latent Diffusion Model Training

To train the LDM in our study, the data for which segmentations were available was randomly split by 80% to 20% into a training dataset and an independent validation dataset. Independence of the datasets was achieved by ensuring that repeated examinations of the same patient were grouped into the same dataset split. During the training of the model, the denoising process from random noise to the final image prediction was subdivided into 1000 steps. The segmentation masks were provided during the training to the network as conditioning with the same size as the latent dimension, which was achieved via down-sampling.

All NN trainings were performed on an NVIDIA DGX workstation utilizing one Tesla V100 graphics processing unit with 32GB memory and an Intel® Xeon® CPU E5-2698 v4 @2.20GHz (20 cores) with 256GB RAM.

S4: Statistical Analysis

Graphics were generated using the `ggplot2` R package, version 3.4.0[6] and the `ggpubr` R package, version 0.5.0[7]. Receiver operating characteristics (ROC) curves were computed with the R package `precrec`, version 0.12.9[8]. DeLong's test[9] to compare two ROC-curves was computed with the R package `pROC`, version 1.18.0[10,11].

S5: Latent Diffusion Model: Conditioning Loss Curves

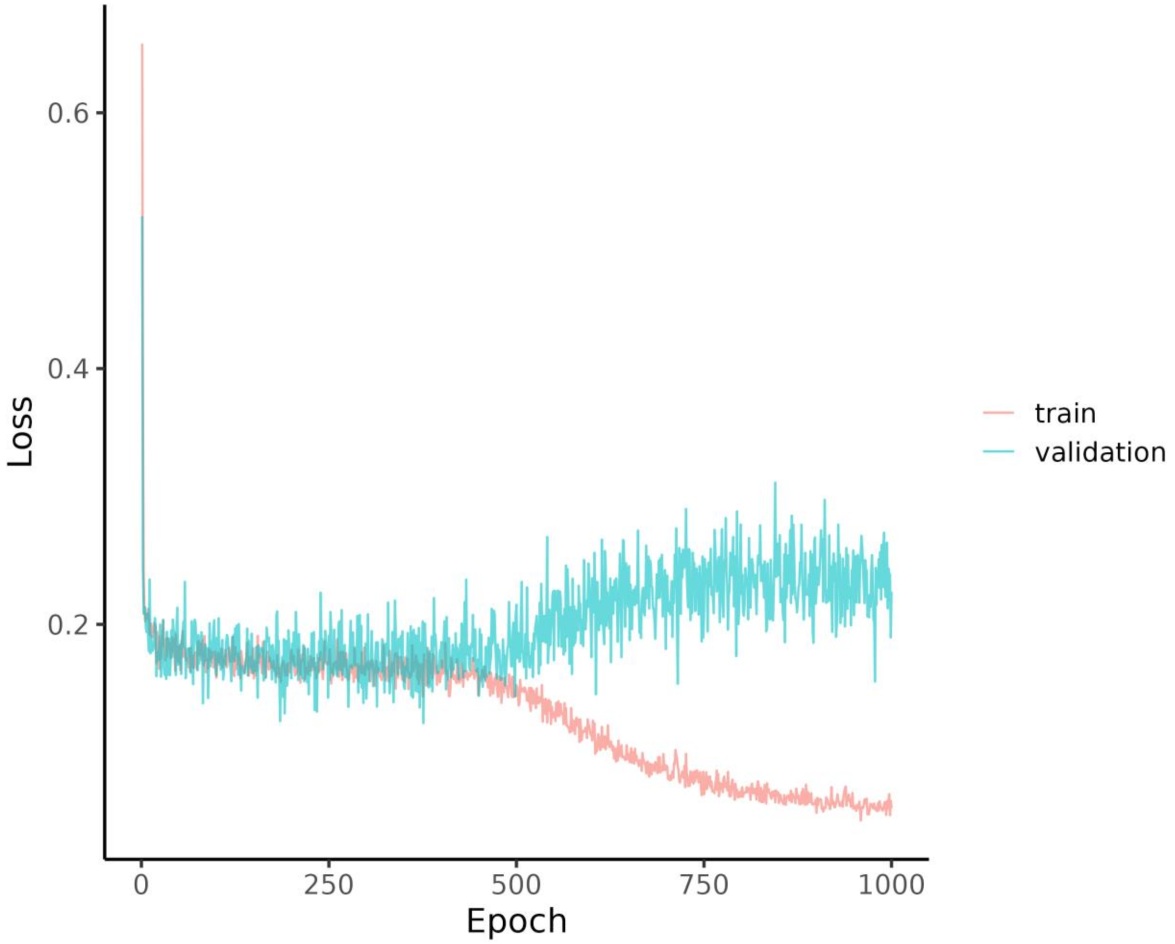

Figure Supp. 1: Training- and validation loss curves of the conditioned latent diffusion model. *The model weights from the epoch with the lowest validation loss (epoch 376) were used to generate the synthetic breast MRI CE-MIPs.*

S6: Interrater Agreement Between Individual Raters

Table Supp. 2: Comparison between readers in BI-RADS scoring (task 1). *W: Kendall's coefficient of concordance. BI-RADS: breast imaging reporting and data system.*

	**Real MIPs**		**Synthetic MIPs**	
**Comparison**	**Kendall's W**	**p-value**	**Kendall's W**	**p-value**
Rater 1 vs. 2	0.79	<0.001	0.848	<0.001
Rater 1 vs. 3	0.893	<0.001	0.921	<0.001
Rater 2 vs. 3	0.79	<0.001	0.884	<0.001

Table Supp. 3: Comparison between readers in scoring any lesions (>= BI-RADS 2) (label derived from task 1). *BI-RADS: breast imaging reporting and data system.*

	**Real MIPs**		**Synthetic MIPs**	
**Comparison**	**Fleiss' Kappa**	**p-value**	**Fleiss' Kappa**	**p-value**
Rater 1 vs. 3	0.113	0.254	0.386	<0.001
Rater 1 vs. 2	0.185	0.062	0.147	0.138
Rater 2 vs. 3	0.025	0.800	0.186	0.060

Table Supp. 4: Comparison between readers in scoring potentially significant lesions (>= BI-RADS 3) (label derived from task 1). *BI-RADS: breast imaging reporting and data system.*

	**Real MIPs**		**Synthetic MIPs**	
**Comparison**	**Fleiss' Kappa**	**p-value**	**Fleiss' Kappa**	**p-value**
Rater 1 vs. 3	0.567	<0.001	0.685	<0.001
Rater 1 vs. 2	0.506	<0.001	0.704	<0.001
Rater 2 vs. 3	0.427	<0.001	0.626	<0.001

Table Supp. 5: Comparison between readers in scoring suspicious lesions (>= BI-RADS 4) (label derived from task 1). *BI-RADS: breast imaging reporting and data system.*

	**Real MIPs**		**Synthetic MIPs**	
**Comparison**	**Fleiss' Kappa**	**p-value**	**Fleiss' Kappa**	**p-value**
Rater 1 vs. 3	0.642	<0.001	0.818	<0.001
Rater 1 vs. 2	0.529	<0.001	0.657	<0.001
Rater 2 vs. 3	0.485	<0.001	0.759	<0.001

Table Supp. 6: Comparison between readers in detecting synthetic MIPs (task 2). *MIP: maximum intensity projection.*

	**Real MIPs**		**Synthetic MIPs**	
**Comparison**	**Fleiss' Kappa**	**p-value**	**Fleiss' Kappa**	**p-value**
Rater 1 vs. 2	0.602	<0.001	0.49	<0.001
Rater 1 vs. 3	-0.336	<0.001	-0.125	0.206
Rater 1 vs. 4	-0.021	0.835	-0.184	0.063
Rater 1 vs. 5	-0.248	0.012	-0.224	0.024
Rater 2 vs. 3	-0.288	0.004	-0.159	0.109
Rater 2 vs. 4	0.133	0.180	-0.087	0.380
Rater 2 vs. 5	-0.202	0.041	-0.126	0.203
Rater 3 vs. 4	-0.105	0.290	-0.042	0.674
Rater 3 vs. 5	-0.181	0.068	-0.005	0.962
Rater 4 vs. 5	0.171	0.083	0.087	0.381

Table Supp. 7: Comparison between readers in scoring anatomical correctness (task 3).

W: Kendall's coefficient of concordance.

	**Real MIPs**		**Synthetic MIPs**	
**Comparison**	**Kendall's W**	**p-value**	**Kendall's W**	**p-value**
Rater 1 vs. 2	0.691	0.007	0.618	0.053
Rater 1 vs. 3	0.621	0.051	0.582	0.124
Rater 1 vs. 4	0.573	0.150	0.486	0.560
Rater 1 vs. 5	0.507	0.442	0.527	0.335
Rater 2 vs. 3	0.624	0.046	0.545	0.252
Rater 2 vs. 4	0.515	0.399	0.503	0.467
Rater 2 vs. 5	0.516	0.395	0.533	0.309
Rater 3 vs. 4	0.582	0.124	0.496	0.507
Rater 3 vs. 5	0.537	0.286	0.434	0.823
Rater 4 vs. 5	0.6	0.084	0.523	0.358

Table Supp. 8: Comparison between readers in scoring realistic image impression (task 4). *W: Kendall's coefficient of concordance.*

	**Real MIPs**		**Synthetic MIPs**	
**Comparison**	**Kendall's W**	**p-value**	**Kendall's W**	**p-value**
Rater 1 vs. 2	0.579	0.133	0.604	0.076
Rater 1 vs. 3	0.443	0.785	0.51	0.424
Rater 1 vs. 4	0.597	0.089	0.441	0.796
Rater 1 vs. 5	0.647	0.025	0.458	0.715
Rater 2 vs. 3	0.548	0.240	0.494	0.516
Rater 2 vs. 4	0.465	0.676	0.527	0.338
Rater 2 vs. 5	0.453	0.738	0.414	0.895
Rater 3 vs. 4	0.587	0.111	0.472	0.639
Rater 3 vs. 5	0.591	0.102	0.601	0.081
Rater 4 vs. 5	0.73	0.002	0.575	0.143

S7: Conditioning Evaluation Stratified by Individual Raters

For rater 1, the area under the receiver operating characteristic curve (AUC) for the detection of any lesions (BI-RADS ≥ 2) in real MIPs was 0.64, whereas in synthetic MIPs the AUC was 0.58 (Figure Supp. 2, R1). With DeLong's test for two ROC curves, no significant differences could be observed between the detection of any lesions in real and synthetic images (p=0.382). Regarding potentially significant lesions (BI-RADS ≥ 3), the AUC in real MIPs was 0.7, whereas in synthetic MIPs the AUC was 0.81 (Figure Supp. 3, R1). With DeLong's test for two ROC curves, no significant differences could be observed between the detection of potentially significant lesions in real and synthetic images (p=0.071). Regarding suspicious lesions (BI-RADS ≥ 4), the AUC in real MIPs was 0.75, whereas in synthetic MIPs the AUC was 0.83 (Figure Supp. 4, R1). With DeLong's test for two ROC curves, no significant differences could be observed between the detection of suspicious lesions in real and synthetic images (p=0.159).

For rater 2, the AUC for the detection of any lesions (BI-RADS ≥ 2) in real MIPs was 0.58, whereas in synthetic MIPs the AUC was 0.57 (Figure Supp. 2, R2). With DeLong's test for two ROC curves, no significant differences could be observed between the detection of any lesions in real and synthetic images (p=0.849). Regarding potentially significant lesions (BI-RADS ≥ 3) the AUC in real MIPs was 0.67, whereas in synthetic MIPs the AUC was 0.78 (Figure Supp. 3, R2). With DeLong's test for two ROC curves, no significant differences could be observed between the detection of potentially significant lesions in real and synthetic images (p=0.082). Regarding suspicious lesions (BI-RADS ≥ 4), the AUC in real MIPs was 0.67, whereas in synthetic MIPs the AUC was 0.78 (Figure Supp. 4, R2). With DeLong's test for two ROC curves, no significant differences could be observed between the detection of suspicious lesions in real and synthetic images (p=0.055).

For rater 3, the AUC for the detection of any lesions (BI-RADS ≥ 2) in real MIPs was 0.55, whereas in synthetic MIPs the AUC was 0.55 (Figure Supp. 2, R3). With DeLong's test for two ROC curves, no significant differences could be observed between the detection of any lesions in real and synthetic images (p=0.961). Regarding potentially significant lesions (BI-RADS ≥ 3) the AUC in real MIPs was 0.77, whereas in synthetic MIPs the AUC was 0.78 (Figure Supp. 3, R3). With DeLong's test for two ROC curves, no significant differences could be observed between the detection of potentially significant lesions in real and synthetic images (p=0.858). Regarding suspicious lesions (BI-RADS ≥ 4), the AUC in real MIPs was 0.69, whereas in synthetic MIPs the AUC was 0.86 (Figure Supp. 4, R3). With DeLong's test for two ROC curves, significant differences could be observed between the detection of suspicious lesions in real and synthetic images (p=0.001).

At this point should be noted that the ground truth of the binary outcomes *potentially significant lesions* and *suspicious lesions* in the reading study dataset differed by only six cases (three cases in each of the synthetic and the real MIPs). This is because only three cases with a BI-RADS score 3 were available in the validation dataset. Clinically, BI-RADS 3 indicates a lesion with a high probability for being benign, however, with the need for a clinical reevaluation after 6 months. From the reading results stratified by the individual raters (see Table Supp. 9) can be seen that raters R1 and R2 mostly avoided to rate MIPs as BI-RADS 3 whereas rater R3 rated 30 real and 19 synthetic MIPs as such.

Figure Supp. 5 shows the per rater results of the assessment of lesions according to BI-RADS (task 1) visualized with boxplots.

The contingency tables with the reading results of the individual raters are given in Table Supp. 9 to Table Supp. 12.

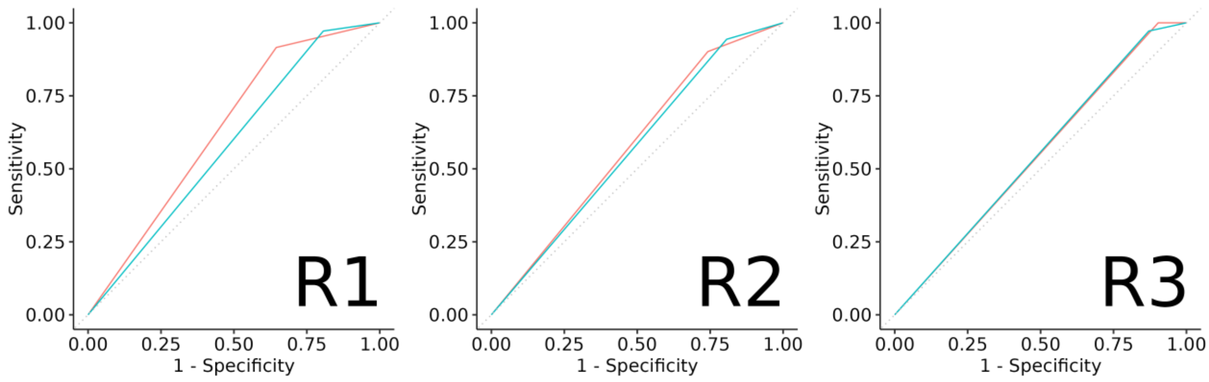

Figure Supp. 2: Receiver operating characteristic (ROC) curves: detection of any lesions (BI-RADS >= 2). *R: Rater. Red line: real images. Blue line: synthetic images. BI-RADS: breast imaging reporting and data system.*

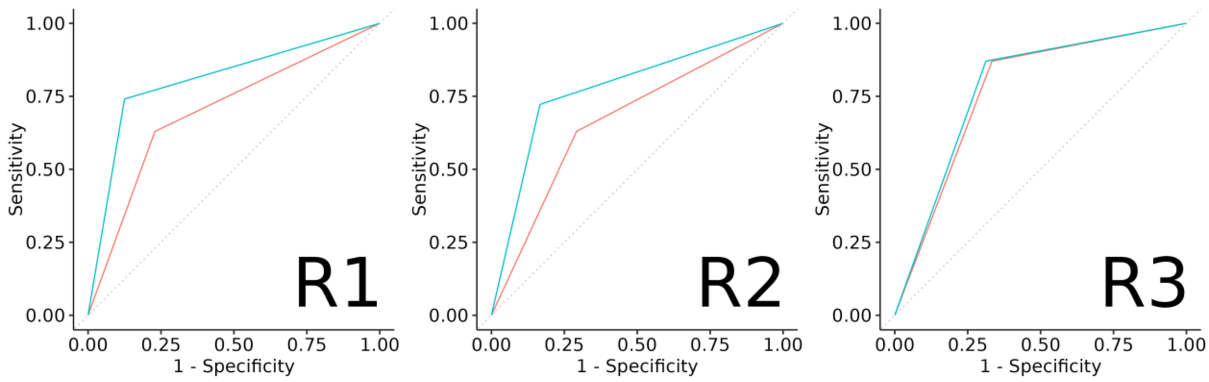

Figure Supp. 3: Receiver operating characteristic (ROC) curves: detection of potentially significant lesions (BI-RADS >= 3). *R: Rater. Red line: real images. Blue line: synthetic images. BI-RADS: breast imaging reporting and data system.*

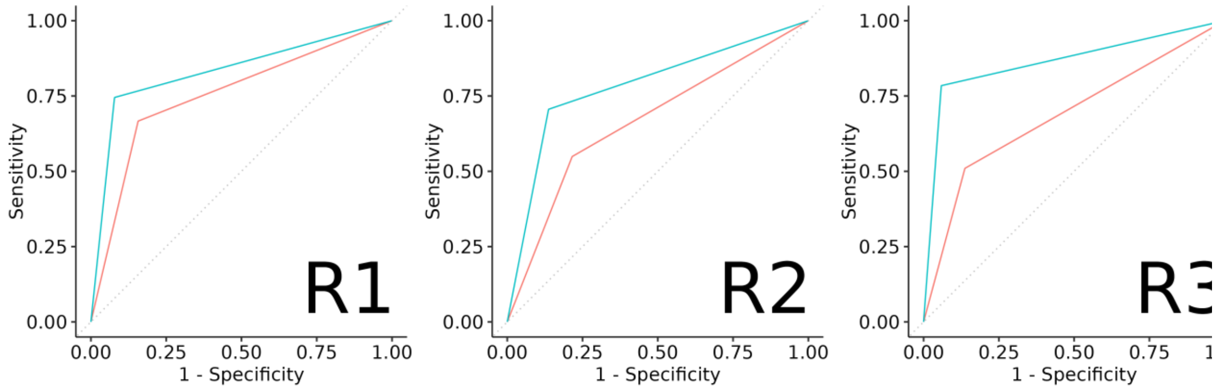

Figure Supp. 4: Receiver operating characteristic (ROC) curves: detection of suspicious lesions (BI-RADS >= 4). *R: Rater. Red line: real images. Blue line: synthetic images. BI-RADS: breast imaging reporting and data system.*

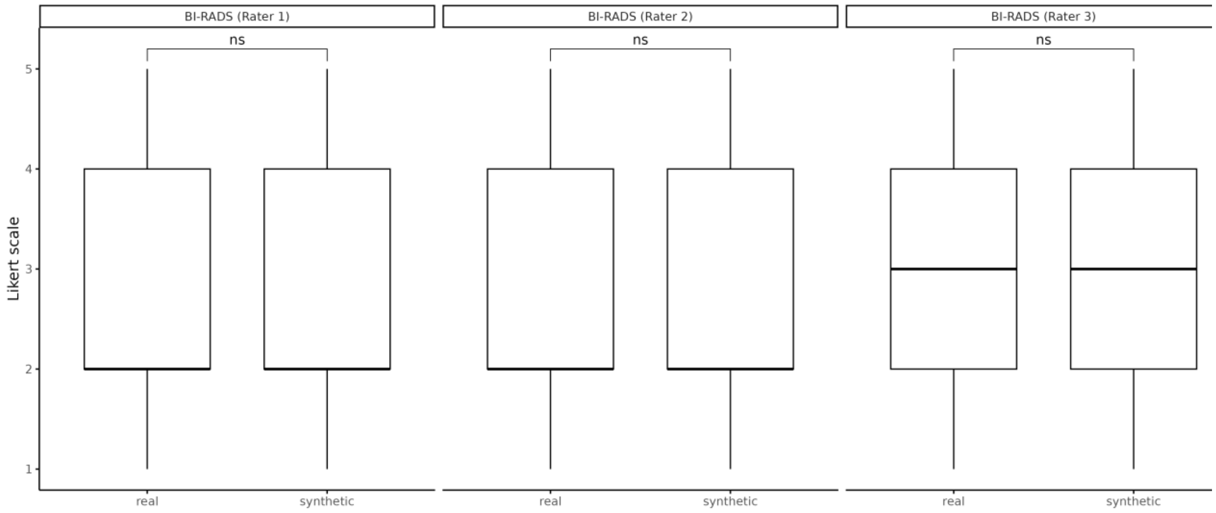

Figure Supp. 5: Boxplots of the per rater label of the lesion assessment according to BI-RADS (task 1). *BI-RADS: breast imaging reporting and data system. MIP: maximum intensity projection. ns: not significant.*

Table Supp. 9: Confusion matrix for each reader's lesion assessment according to BI-RADS for real and synthetic MIPs. *BI-RADS: breast imaging reporting and data system. GT: ground truth. MIP: maximum intensity projection.*

**Rater**		**Reading**	**GT 1**	**GT 2**	**GT 3**	**GT 4**	**GT 5/6**	**Total**
R1	Real MIPs	1	11	2	1	2	1	17
		2	16	8	2	6	8	40
		3	1	2	0	0	0	3
		4	2	5	0	9	6	22
		5	1	0	0	1	18	20
		Total	31	17	3	18	33	102
	Synthetic MIPs	1	6	1	0	1	0	8
		2	22	13	3	6	4	48
		3	1	1	0	0	2	4
		4	2	2	0	7	15	26
		5	0	0	0	4	12	16
		Total	31	17	3	18	33	102
R2	Real MIPs	1	8	3	0	1	3	15
		2	18	5	2	7	7	39
		3	2	2	0	4	1	9
		4	3	7	1	6	11	28
		5	0	0	0	0	11	11
		Total	31	17	3	18	33	102
	Synthetic MIPs	1	6	4	0	0	0	10
		2	22	8	2	8	5	45

		3	2	0	0	2	0	4
		4	1	4	0	4	12	21
		5	0	1	1	4	16	22
		Total	31	17	3	18	33	102
R3	Real MIPs	1	3	0	0	0	0	3
		2	21	8	2	3	2	36
		3	2	7	1	13	7	30
		4	5	2	0	2	15	24
		5	0	0	0	0	9	9
		Total	31	17	3	18	33	102
	Synthetic MIPs	1	4	2	0	0	0	6
		2	20	7	1	5	1	34
		3	7	5	2	3	2	19
		4	0	3	0	10	26	39
		5	0	0	0	0	4	4
		Total	31	17	3	18	33	102

Table Supp. 10: Confusion matrix for each reader's scoring regarding any lesions (>= BI-RADS 2) for real and synthetic MIPs. *BI-RADS: breast imaging reporting and data system. GT: ground truth. 0: no lesion. 1: any lesion. MIP: maximum intensity projection.*

**Rater**		**Reading**	**GT 0**	**GT 1**	**Total**
R1	Real MIPs	0	11	6	17
		1	20	65	85
		Total	31	71	102
	Synthetic MIPs	0	6	2	8
		1	25	69	94
		Total	31	71	102
R2	Real MIPs	0	8	7	15
		1	23	64	87
		Total	31	71	102
	Synthetic MIPs	0	6	4	10
		1	25	67	92
		Total	31	71	102
R3	Real MIPs	0	3	0	3
		1	28	71	99
		Total	31	71	102
	Synthetic MIPs	0	4	2	6
		1	27	69	96
		Total	31	71	102

Table Supp. 11: Confusion matrix for each reader's scoring regarding potentially significant lesions (>= BI-RADS 3) for real and synthetic MIPs. *BI-RADS: breast imaging reporting and data system. GT: ground truth. 0: not significant lesion. 1: potentially significant lesion. MIP: maximum intensity projection.*

**Rater**		**Reading**	**GT 0**	**GT 1**	**Total**
R1	Real MIPs	0	37	20	57
		1	11	34	45
		Total	48	54	102
	Synthetic MIPs	0	42	14	56
		1	6	40	46
		Total	48	54	102
R2	Real MIPs	0	34	20	54
		1	14	34	48
		Total	48	54	102
	Synthetic MIPs	0	40	15	55
		1	8	39	47
		Total	48	54	102
R3	Real MIPs	0	32	7	39
		1	16	47	63
		Total	48	54	102
	Synthetic MIPs	0	33	7	40
		1	15	47	62
		Total	48	54	102

Table Supp. 12: Confusion matrix for each reader's scoring regarding suspicious lesions (>= BI-RADS 4) for real and synthetic MIPs. *BI-RADS: breast imaging reporting and data system. GT: ground truth. 0: not suspicious. 1: suspicious lesion. MIP: maximum intensity projection.*

**Rater**		**Reading**	**GT 0**	**GT 1**	**Total**
R1	Real MIPs	0	43	17	60
		1	8	34	42
		Total	51	51	102
	Synthetic MIPs	0	47	13	60
		1	4	38	42
		Total	51	51	102
R2	Real MIPs	0	40	23	63
		1	11	28	39
		Total	51	51	102
	Synthetic MIPs	0	44	15	59
		1	7	36	43
		Total	51	51	102
R3	Real MIPs	0	44	25	69
		1	7	26	33
		Total	51	51	102
	Synthetic MIPs	0	48	11	59
		1	3	40	43
		Total	51	51	102

S8: Detection of Synthetic Images Stratified by Raters

For rater 1, the AUC for the detection of synthetic images was 0.55 (Figure Supp. 6, R1). For rater 2, the AUC for the detection of synthetic images was 0.53 (Figure Supp. 6, R2). For rater 3, the AUC for the detection of synthetic images was 0.58 (Figure Supp. 6, R3). For rater 4, the AUC for the detection of synthetic images was 0.64 (Figure Supp. 6, R4). For rater 5, the AUC for the detection of synthetic images was 0.51 (Figure Supp. 6, R5).

The contingency tables with the reading results of the individual raters are given in Table Supp. 13.

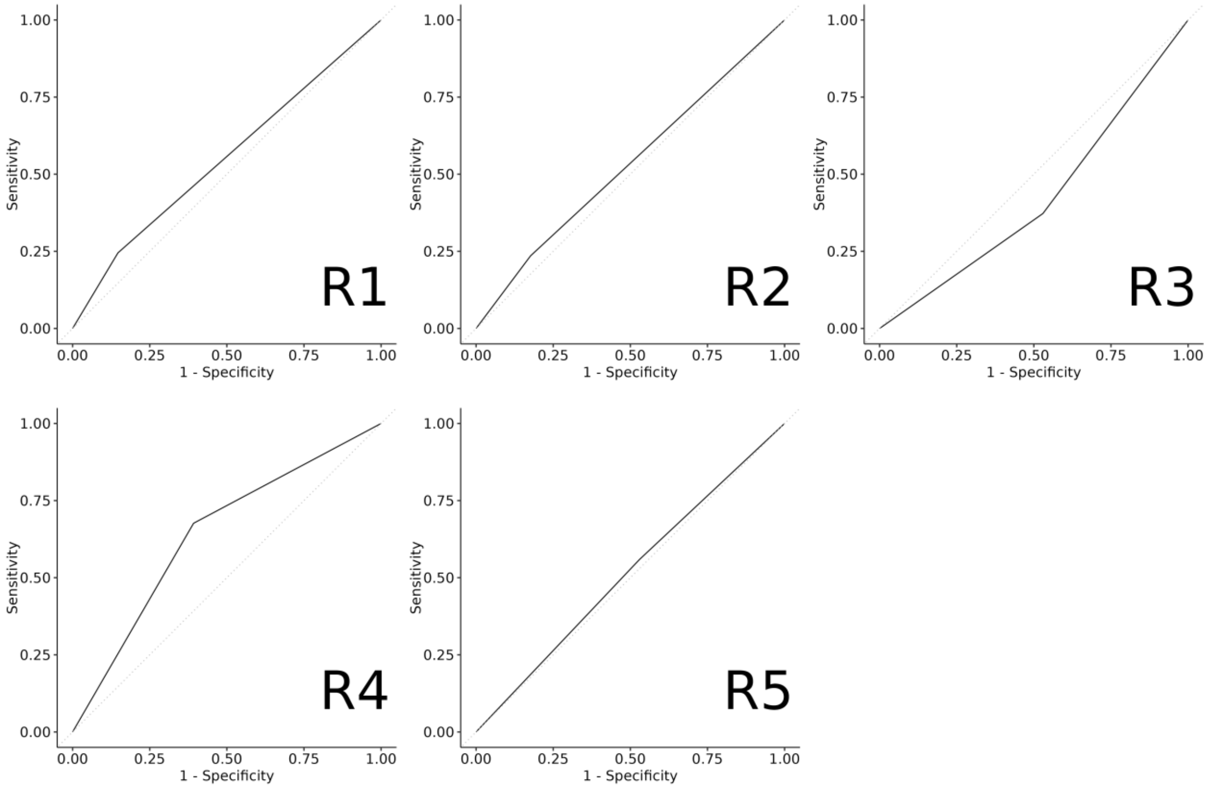

Figure Supp. 6: Receiver operating characteristic (ROC) curves: detection of synthetic maximum intensity projections. *R: Rater.*

Table Supp. 13: Confusion matrix of each reader's results of detecting synthetic MIPs.

GT: ground truth. 0: MRI-acquired MIP. 1: synthetic MIP. MIP: maximum intensity projection.

**Rater**	**Reading**	**GT 0**	**GT 1**	**Total**
R1	0	87	77	164
	1	15	25	40
	Total	102	102	204
R2	0	84	78	162
	1	18	24	42
	Total	102	102	204
R3	0	48	64	112
	1	54	38	92
	Total	102	102	204
R4	0	62	33	95
	1	40	69	109
	Total	102	102	204
R5	0	48	45	93
	1	54	57	111
	Total	102	102	204

S9: Anatomical Correctness and Realistic Image Impression

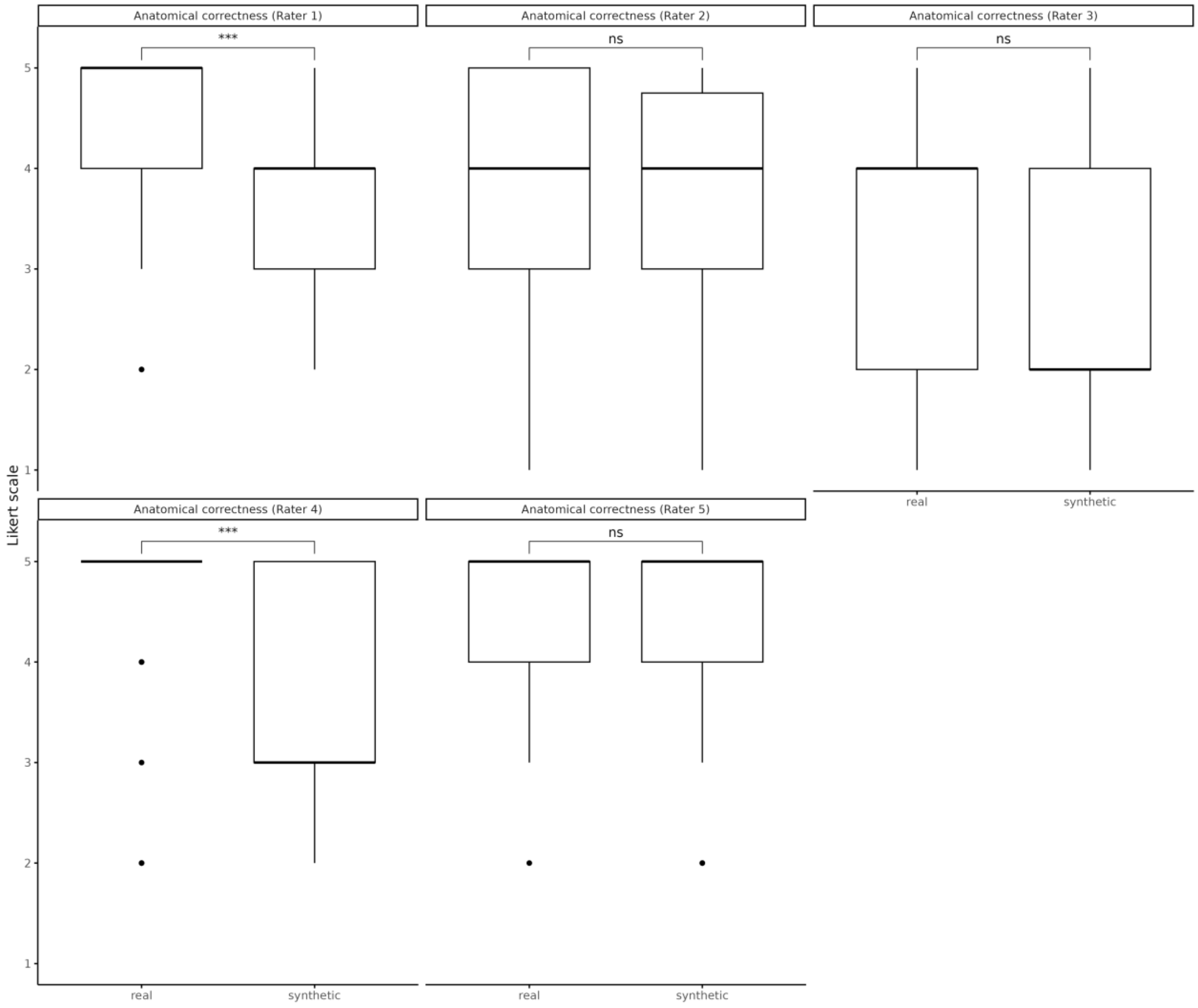

Figure Supp. 7: Boxplots of the per rater label of the Likert-scaled reading regarding 'anatomical correctness' (reading task 3). *Likert-scale: 1- strongly disagree; 2-disagree; 3-neither agree nor disagree; 4-agree; 5-strongly agree. ns: not significant. ***: p-value of the Wilcoxon Rank Sum Test < 0.001.*

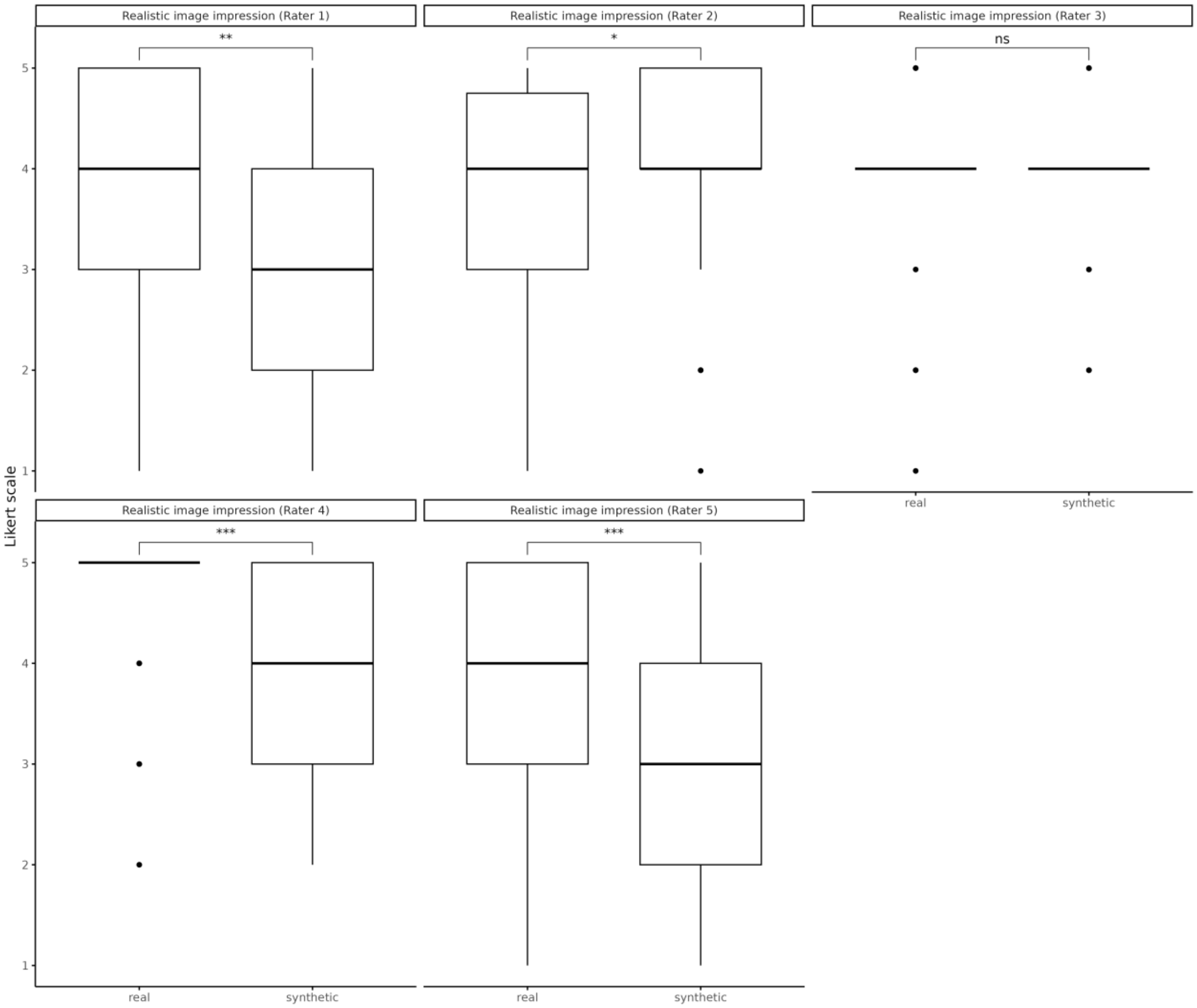

Figure Supp. 8: Boxplots of the per rater label of the Likert-scaled reading regarding 'realistic image impression' (reading task 4). *Likert-scale: 1- strongly disagree; 2-disagree; 3-neither agree nor disagree; 4-agree; 5-strongly agree. ns: not significant. Wilcoxon Rank Sum Test p-values: * = < 0.05; ** = < 0.01; *** = < 0.001.*

Table Supp. 14: Confusion matrix of each reader's results of evaluation of the anatomical correctness (reading task 3). *GT: ground truth.*

Rater	Score	GT real	GT synthetic	Total
R1	1 - strongly disagree	0	0	0
	2 - disagree	4	6	10
	3 - neither agree nor disagree	11	24	35
	4 - agree	30	53	83
	5 - strongly agree	57	19	76
	Total	102	102	204
				Fisher's p=0.000
R2	1 - strongly disagree	5	4	9
	2 - disagree	10	15	25
	3 - neither agree nor disagree	16	21	37
	4 - agree	31	36	67
	5 - strongly agree	40	26	66
	Total	102	102	204
				Fisher's p=0.282
R3	1 - strongly disagree	6	2	8
	2 - disagree	39	54	93
	3 - neither agree nor disagree	3	11	14
	4 - agree	49	33	82
	5 - strongly agree	5	2	7
	Total	102	102	204
				Fisher's p=0.008

R4	1 - strongly disagree	0	0	0
	2 - disagree	8	23	31
	3 - neither agree nor disagree	2	30	32
	4 - agree	12	19	31
	5 - strongly agree	80	30	110
	Total	102	102	204
				Fisher's p=0.000
R5	1 - strongly disagree	0	0	0
	2 - disagree	2	5	7
	3 - neither agree nor disagree	9	5	14
	4 - agree	26	40	66
	5 - strongly agree	65	52	117
	Total	102	102	204
				Fisher's p=0.075

Table Supp. 15: Confusion matrix of each reader's results of evaluation of the realistic image impression (reading task 4). *GT: ground truth.*

Rater	Score	GT real	GT synthetic	Total
R1	1 - strongly disagree	7	3	10
	2 - disagree	18	36	54
	3 - neither agree nor disagree	15	18	33
	4 - agree	27	29	56
	5 - strongly agree	35	16	51
	Total	102	102	204
				p=0.005 (Chi-squared test)
R2	1 - strongly disagree	4	1	5
	2 - disagree	12	11	23
	3 - neither agree nor disagree	26	12	38
	4 - agree	34	40	74
	5 - strongly agree	26	38	64
	Total	102	102	204
				Fisher's p=0.042
R3	1 - strongly disagree	2	0	2
	2 - disagree	10	13	23
	3 - neither agree nor disagree	2	4	6
	4 - agree	66	69	135
	5 - strongly agree	22	16	38
	Total	102	102	204
				Fisher's p=0.450

R4	1 - strongly disagree	0	0	0
	2 - disagree	3	11	14
	3 - neither agree nor disagree	8	27	35
	4 - agree	12	34	46
	5 - strongly agree	79	30	109
	Total	102	102	204
				Fisher’s p=0.000
R5	1 - strongly disagree	3	3	6
	2 - disagree	10	31	41
	3 - neither agree nor disagree	14	19	33
	4 - agree	26	31	57
	5 - strongly agree	49	18	67
	Total	102	102	204
				Fisher’s p=0.000

S10: Schema of Visual Appearance of Lesions

We assume that there is some heterogeneity in the visual appearance of lesions of a specific BI-RADS class on CE-MIPs, which is likely following a normal distribution (see schema in Figure Supp. 9) and when considering only MIPs, overlaps might occur between the visual appearance of lesions of different classes. The ground truth of the lesions depicted on the MIPs, however, was established using the clinical reports, which were based on the full diagnostic multiparametric protocol and which contained much more information than visible in the MIPs. As only the segmentation masks (consisting of information on the location of lesions and their underlying BI-RADS score) were provided as conditions during the training of the LDM, the additional information from the clinical reports and other MRI sequences was naturally lacking. Thus, based on the available information, the NN might have inferred general patterns between heterogeneous appearing lesions of the same class with a higher confidence, reflected by the non-overlapping regions in the schematic distribution curves of the lesion appearance in Figure Supp. 9. When applying the LDM to generate synthetic data, these patterns may be reflected insofar as the NN could tend to generate lesions that can be assigned more clearly to a particular class (in this case the BI-RADS score), which we refer to as 'typical' or 'textbook representations'.

Future studies should investigate, if this could be overcome so that also edge cases are represented better in the synthetic data. For example, additionally to the segmented lesions, trainings could be conditioned by confidence measures, which could for example be established by incorporating the degree of agreement between multiple raters. Another approach could include to subdivide established classes into more fine granular segments which can be used for conditioning.

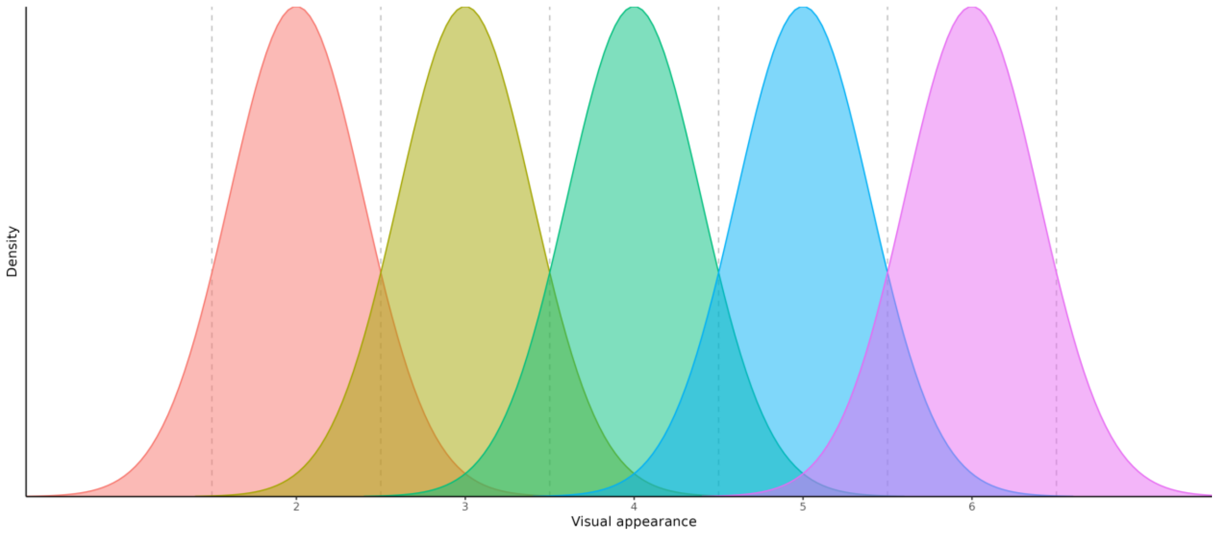

Figure Supp. 9: Schema of the visual appearance of lesions on CE-MIPs. *The x-axis shows the class of the lesion according to BI-RADS. BI-RADS: breast imaging reporting and data system. CE: contrast enhanced. MIP: maximum intensity projection.*

References

1. Kapsner, L. A. *et al.* Automated artifact detection in abbreviated dynamic contrast-enhanced (DCE) MRI-derived maximum intensity projections (MIPs) of the breast. *European Radiology* **32**, 5997–6007 (2022).

2. Lowekamp, B. C., Chen, D. T., Ibanez, L. & Blezek, D. The Design of SimpleITK. *Front. Neuroinform.* (2013) doi:10.3389/fninf.2013.00045.

3. Yaniv, Z., Lowekamp, B. C., Johnson, H. J. & Beare, R. SimpleITK Image-Analysis Notebooks: A Collaborative Environment for Education and Reproducible Research. *J Digit Imaging* **31**, 290–303 (2018).

4. Marcus, D. S., Olsen, T. R., Ramaratnam, M. & Buckner, R. L. The extensible neuroimaging archive toolkit: An informatics platform for managing, exploring, and sharing neuroimaging data. *Neuroinformatics* **5**, 11–33 (2007).

5. Harris, C. R. *et al.* Array programming with NumPy. *Nature* **585**, 357–362 (2020).

6. Wickham, H. ggplot2: Elegant graphics for data analysis. (2016).

7. Kassambara, A. Ggpubr: 'ggplot2' based publication ready plots. (2022).

8. Saito, T. & Rehmsmeier, M. Precrec: Fast and accurate precision-recall and ROC curve calculations in r. **33 (1)**, 145–147 (2017).

9. DeLong, E. R., DeLong, D. M. & Clarke-Pearson, D. L. Comparing the areas under two or more correlated receiver operating characteristic curves: A nonparametric approach. *Biometrics* **44**, 837 (1988).

10. Robin, X. *et al.* pROC: An open-source package for r and s+ to analyze and compare ROC curves. **12**, 77 (2011).

11. Robin, X. *et al.* pROC: an open-source package for R and S+ to analyze and compare ROC curves. *BMC Bioinformatics* **12**, (2011).
